# Macrophages and brown adipocytes cross-communicate to modulate a thermogenic program following methamphetamine exposure

**DOI:** 10.1080/02656736.2020.1849822

**Published:** 2020

**Authors:** Manuel Sanchez-Alavez, Nikki Bortell, Liana Basova, Fahumiya Samad, Maria Cecilia Garibaldi Marcondes

**Affiliations:** a Department of Neuroscience, The Scripps Research Institute, La Jolla, CA, USA; b Facultad de Medicina y Psicología, Universidad Autónoma de Baja California, Tijuana, México; c San Diego Biomedical Research Institute, San Diego, CA, USA

**Keywords:** Methamphetamine, thermogenesis, brown adipocytes, macrophages

## Abstract

Hyperthermia is a potentially lethal side-effect of Methamphetamine (Meth), a stimulant drug. Activation of non-shivering thermogenesis in brown adipose tissue (BAT) is partly responsible for Meth-induced rise in temperature, with contributing sympathetic neurotransmitters, such as norepinephrine (NE), and reactive oxygen species (ROS). However, the mechanisms controlling the development of a molecular thermogenic program in brown adipocytes (BA) following Meth are unknown. We hypothesize that Meth and NE affect BAT cells, BA and macrophages, to modify their physiology and interactions, with consequences to thermogenic genes. We also hypothesize that ROS play a critical role in signaling transcription of thermogenic genes and their regulatory components. Using primary BA and macrophage cultures, we measured Meth and NE interference with physiological and phenotypic measures that are relevant to thermogenesis in BAT. Meth caused both BA and macrophages to decrease mitochondrial maximal capacity and increase ROS. In BA, the thermogenic genes UCP1, PPARγ, PGC1α and GADD45γ were transcriptionally increased by Meth in a ROS-dependent manner. In macrophages, Meth increased oxidative stress response and caused a predominance of M2 subset markers. BA transcriptional changes in response to Meth and NE were significantly controlled by macrophages. The results suggest that BA and macrophages respond to Meth and NE, with effects on mitochondrial functions and transcription of genes involved in thermogenesis. ROS-dependent signals in BA and cellular interactions between BA and macrophages synergize to regulate the BAT environment and control critical pathways leading to Meth-hyperthermia.

## Introduction

Hyperthermia is a potentially lethal side effect of Methamphetamine (Meth), a psychostimulant and drug of addiction, which is highly associated to neurotoxicity and cognitive decay [1–4]. To date, pharmacological approaches to rescue psychostimulant-induced hyperthermia are not available, mainly due to the lack of understanding of mechanisms associated with its development. Imaging technologies have recently demonstrated brown adipose tissue (BAT), a peripheral thermogenic site, as a critical participant in non-shivering thermogenesis in human adults [5]. Our group has demonstrated that BAT is responsible for 40% of the elevation in core body temperature (CBT) that occurs in drug-naïve animals exposed to Meth [6,7]. Denervation of BAT was able to reduce the effect of Meth on CBT, suggesting participation of sympathetic input [7]. The administration of an anti-oxidant, such as N-Acetyl Cysteine (NAC) prior to, or after the injection of Meth, prevented and reverted Meth-induced hyperthermia [7] suggesting that reactive oxygen species (ROS), which are acutely induced by Meth in BAT and elsewhere [6,7], may play an important role in Meth hyperthermia. However, the exact mechanisms leading to changes in thermogenesis in BAT are unknown.

Cellular interactions in BAT can be critical for understanding the thermogenesis process in Meth exposure. For instance, the BAT contains a large population of macrophages, which have been suggested to control innervation, energy expenditure [8], as well as thermogenesis, particularly in conditions such as cold adaptation [9]. Macrophages may interfere with thermogenesis via a Stat-6–dependent M2 phenotype [9], via type 2 cytokines such as IL4 and IL13 [9,10], or through a described ability to produce catecholamines [9]. Meth stimulation does not cause significant changes in IL4 and IL13 [11], nor can induce increases in catecholamine production by macrophages [12]. Yet, the contribution of macrophages to changes in BAT molecular patterns in thermogenesis following Meth exposure remains unknown.

Both brown adipocytes (BA) and macrophages in BAT may be affected during Meth exposure. A major sympathetic neurotransmitter released upon Meth administration is norepinephrine (NE) [13,14], which has been shown to modulate phenotypic and transcriptional patterns associated with thermogenesis BA [15–20]. We hypothesize that Meth and NE interact with cells in BAT, particularly brown adipocytes (BA) and macrophages, to modify their physiology and interactions, with important consequences to the expression of thermogenic genes. We also hypothesized that ROS play a critical role in signaling the transcription of thermogenic genes and their regulatory components. We used *in vitro* approaches, with primary BA and macrophages, to test the ability of Meth and NE to trigger ROS, modify mitochondrial functions, and ultimately phenotypes that are relevant to thermogenesis in BAT. We also characterized the phenotype of BAT-derived macrophages. Finally, using co-cultures of BA and macrophages, we tested the capacity of macrophages to modulate BA response to Meth and NE.

We found that ROS and mitochondrial functions are impacted by Meth in both BA and macrophages, that ROS-dependent signaling may upregulate several thermogenesis-relevant genes, and that macrophages, expressing predominantly M2 markers, can largely control the expression of genes involved in thermogenesis expressed by BA. Our results add up to our understanding of psychostimulant drug-hyperthermia, showing the contribution of molecular and cellular interactions occurring in BAT, and offering novel insights on modulatory prospects to treat life-threatening hyperthermia syndrome in drug abuse [9,21].

## Materials and methods

### Animals

All experiments were approved by the Institutional Animal Care and Use Committees of the San Diego Biomedical Research Institute and The Scripps Research Institute. Newborn C57Bl/6 mice were used to prepare primary cultures of BA, performed as described [22]. Male 6 to 8-weeks-old C57BL/6J mice were used for isolation of macrophages from BAT, and from the peritoneal cavity, as well as for the isolation of these cell types following *in vivo* Meth administration (described below). The isolated cells were used for *in vitro* experiments and analysis.

### In vivo administration of meth

The intraperitoneal administration of 3 mg/kg of (+) Methamphetamine hydrochloride (M8750 – Sigma-Aldrich), or vehicle, was performed in male C57BL/6 mice between 6 and 8-weeks-old, following a previously described protocol [6,7].

### Brown adipocyte primary cultures

As previously described [22], interscapular BAT from 5–7 newborn C57BL/6 mice were dissected, rinsed in PBS, minced, and digested for 40 min at 37 °C in 0.1% collagenase type I (Sigma-Aldrich). Then, it was filtered through a 100-mm nylon mesh to remove undigested tissue and centrifuged at 1000 *g* for 5 min. The pellets were washed in PBS and resuspended in DMEM with 15% FBS (Hyclone) and 1% penicillin/streptomycin (Gibco). Adipocyte differentiation was induced for 48 h, in media containing 5 μg/ml recombinant insulin (R&D Systems), 1 nM 3,3′,5-triiodo-l-thyronine (T3, R&D Systems), 125 μM indomethacin (Sigma-Aldrich), 500 μM iso-butylmethylxanthine (IBMX, Sigma-Aldrich) and 1 μM dexa-methasone (Sigma-Aldrich). The media was refreshed every 2 days. Differentiation phenotype was fully achieved on day 6, when cells presented a BA morphology, with a large accumulation of fat droplets. The assays and co-cultures were performed on 7-day cultures. After the assays, and at time points described in legends, cells were harvested from the cultures and used in molecular studies.

### Macrophages in co-cultures

Macrophages were elicited in the peritoneal cavity of 6–8 weeks old male C57Bl/6 mice, by a 1 ml intraperitoneal injection of sterile 4% thyoglycolate (Sigma-Aldrich), prepared and kept sterile in the dark. Four days after the injection days, macrophages were harvested by peritoneal lavage with 4 ml ice cold PBS. After a wash, the cells were resuspended in DMEM supplemented with 10% FBS and 1% penicillin/streptomycin (Gibco). Macrophages were added to the primary adipocyte cultures at different ratios and let stand overnight prior to the *in vitro* stimulations. Results show the 1:10 macrophage/brown adipocyte ratio.

### In vitro *meth, NE and NAC treatments*

Reagent doses were optimized in dose-response experiments in both macrophages and BAs, to account for viability and biological effects. (+) Methamphetamine hydrochloride (M8750 – Sigma-Aldrich) was added to the culture media at 60 μM, as previously reported [6,7,23] and cells were harvested at indicated time-points. (−) Norepinephrine (A7257, Sigma Aldrich) was added to the cultures at 0.1 μM. N-acetyl cysteine was added at 5 pg/ml (A7250, Sigma Aldrich). ROS measurements were performed at 30 min and 24 h after stimuli. Assays were performed 24 h after stimulations unless stated in figure legends. Trypan blue (Sigma Aldrich) dye exclusion staining was performed to count cells and assess viability upon plating and retrieval. Cultures with less than 80% viability were discarded.

### Gene expression assays

Total RNA was purified from samples using RNA kit (Thermo-Fisher) following the manufacturer’s protocol. RNA was further purified by RNeasy mini kit (Qiagen, Valencia, CA). The cDNA was obtained using RT2 First-strand kits (Qiagen) following the manufacturer’s instructions. PCR reactions were performed in an ABI 7500 real-time thermal cycler (Applied Biosystems), run by Melt High Resolution (HRM) software, using SyBrGreen ROX Mastermix (Qiagen). The changes in genes involved in the oxidative stress response were measured in macrophages isolated from BAT, and stimulated with Meth or NE, as above, using RT^2^ Profiler™ PCR Array Mouse Oxidative Stress and Antioxidant Defense (Qiagen, Cat No. 330231). Primers in Table 1 were purchased from Qiagen. The expression was normalized by an average of GAPDH and 18S expression. Results were expressed as log 2 ddCT relative values.

### Visualization of transcriptional changes in functional gene clusters

Visualization of the effects of Meth and NE in macrophage gene clusters was performed in Cytoscape (http://www.cyto-scape.org/) [24,25] using GeneMania (http://www.genemania.org/) [26–29] with ddCT, calculated fold change and p values imported as metadata in .txt, and loaded as node attributes, as previously described by us [30,31].

### Immunodetection of CD68+ macrophages in BAT

Upon dissection, the harvested tissue was embedded in Tissue-Tek OCT compound (Sakura Finetech, VWR, Radnor, PA), slowly frozen on dry ice, and maintained at −80 °C until use. Primary labeling was performed on sections with pretitrated dilutions of Alexafluor 488-labelled anti-rat polyclonal antibody (Biolegend) using standard procedures, as described by us [6]to verify the morphology and distribution of the macrophage marker CD68. For that, 30-micron sections were air-dried in the dark and then incubated in 10% normal goat serum (Vector) for 60 min at room temperature. Tissue was permeabilized with 0.2% Triton X-100 and 0.1 M-glycine in PBS for 7 min at room temperature. Between staining steps, segments were washed in PBS containing 0.1% Tween-20. Each section was placed on a glass slide, mounted, and pressed overnight. Sample visualization was performed with a confocal microscope (2100 Radiance; BioRad Laboratories). The 40× and 63× objectives were used to collect high-resolution images with an x–y resolution of 0.15 μm and a z-distance resolution of 0.80 μm. To characterize cells, z-series optical sectioning was performed with the 40× or 63× objectives, creating image stacks that span the thickness of the total section. z-Series image stacks were collected at 0.5 μm z-distance increments. In the z-series images, green autofluorescence and background were sorted from bright staining. Three-dimensional analysis of regions of interest was performed on ImageJ (National Institutes of Health) and Zen Microscope software (Zeiss, Peabody, MA).

### Amplex Red assay for hydrogen peroxide detection in culture supernatants

The H_2_O_2_ production levels that resulted from Meth or NE in aqueous solution were determined by real-time fluorometric detection of H_2_O_2_ using an Amplex Red (Invitrogen) assay in an Infinite F500 luminometer (Tecan Systems). The addition of 2 U/ml Catalase (C1345, Sigma-Aldrich) confirmed hydrogen peroxide production.

### Dihydroethidium (DHE) staining and quantification

A 5 μM DHE staining solution was freshly prepared prior to use, from a 10mM stock solution in DMSO. For that, 25 μl DHE stock solution was diluted in 50 ml Milli-Q pure H_2_O. After aspirating most of the media, and 130 ul of DHE staining solution was placed in each culture well and incubated for 20 min at room temperature and away from the light. The cells were washed twice in deionized water for 1 min. Fluorescence imaging was performed immediately using an Axiovert 200 inverted microscope (Carl Zeiss) with Axio Vision software (version 4.8.1; Carl Zeiss). Fluorescence intensity was calculated in Fiji/ImageJ (National Institute of Health, USA). For that, tiff image files were transformed in 8-bit, and a manually set threshold was set to identify stained cells in control specimens, and then applied to all compared images. Measurement values were normalized to the area, in a minimum of six 60x magnification images for each condition in triplicate, and averaged.

### JC-1 assay for mitochondria membrane potential

To assess relative changes in mitochondrial membrane potential during development, cells were incubated with 1.5M JC-1 Mito Probe (Molecular Probes) following the manufacturer’s protocols. JC-1 aggregate (red) and JC-1 monomer (green) fluorescence wavelengths were measured simultaneously for 30 min using a Tecan Infinite F500 apparatus (Tecan Systems). JC-1 aggregates were detected at 535-nm excitation and 590-nm emission, and monomers were detected at 485-nm excitation and 535-nm emission. Positive (rotenone) and negative controls (buffer), and microscopic examination, were used for relative change calculations. Results are expressed in fold differences between monomers and aggregates.

### Mitochondrial respiratory function

The effects of Meth and NE on the mitochondrial oxygen consumption rate (OCR) in the intact primary BA were assessed by using a Seahorse Bioscience XFe96 analyzer (Massachusetts, USA) in combination with the Seahorse Bioscience XF Cell Mito Stress Test assay kit (Agilent), which contains mitochondrial modulating compounds, following manufacturer’s protocols. Compounds used in the assay were the ATP synthase inhibitor oligomycin (2 μM), the mitochondrial uncoupler carbonyl cyanide 4-(trifluoromethoxy) phenyl-hydrazone (FCCP, 0.5 μM) and the complex I and II inhibitors rotenone (1 μM) and antimycin A (1 μM) (RAA), to examine different aspects of mitochondrial function. Briefly, the isolated BA were counted and plated at 10^5^ cells/100 μl/well in a 96-well assay plate (Seahorse Bioscience) in triplicates, as described above. The differentiation medium was replaced by a medium containing Meth or NE, and after 24 h, the normal medium was replaced by an assay medium consisting of XF Base Medium (Seahorse Bioscience) with added 10mM glucose, 10mM pyruvic acid, and 1mM L-glutamine. The analysis of OCR was performed in a Seahorse XFe96 analyzer according to the manufacturer’s instructions. The OCR values were obtained during the baseline for 20 min, and then after the addition of the compounds. Prior to analysis, data were corrected by withdrawing non-mitochondrial respiration (measured after rotenone + antimycin A) from all OCR values.

### Statistical analysis

Experiments were performed in triplicates, and a minimum of two biological replicates. All assays were analyzed using one and two-factor ANOVAs with Bonferroni’s *post hoc* tests, with a significant *p*-value <0.05 between comparisons indicated in figures, using Prism software (Graphpad Software, San Diego, CA).

## Results

### Meth and NE affect macrophages and BA

The cell subset-specific effects of Meth can result from the direct interaction with the drug and from sympathetically (NE)-mediated effects of Meth. NE is delivered to BAT through innervation. We have previously shown that in animals with BAT ablation or denervation, hyperthermia is significantly reduced following Meth [7]. In order to understand the contribution of BAT to Meth thermogenesis, we explored the effects of Meth and NE on the physiology and functions of the two main cell types in BAT: macrophages and BA. We also examined whether these two cell types interact upon the development of transcriptional changes that are associated with hyperthermia in BA.

### Effects of meth and NE on ROS production and mitochondrial functions in BA and macrophages

**Figure 1.**
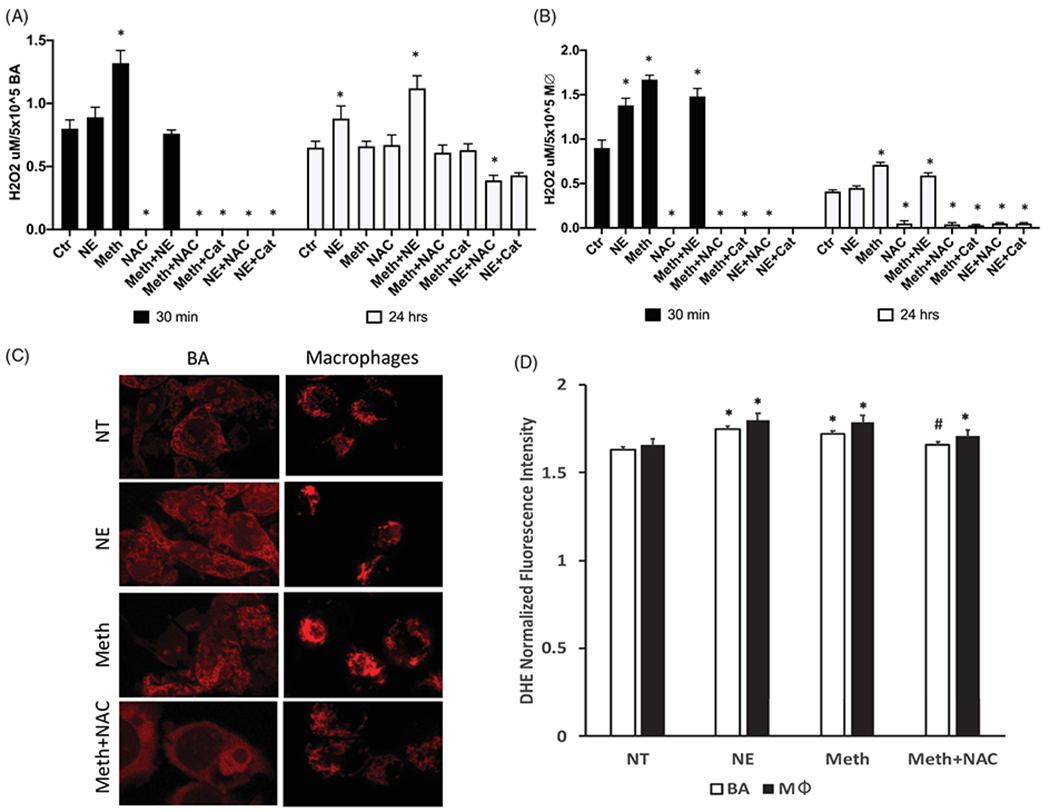
Effects of Meth and NE on ROS production in BA and macrophages. (A) H_2_O_2_ was measured in the supernatant of BA primary cultures 30 min and 24 h following exposure with 0.1 μM NE, 60 μM Meth, and/or 5 pg/ml N-acetyl D-cysteine (NAC), and/or 2 U/ml of Catalase (Cat). (B) H_2_O_2_ was measured in macrophage culture supernatants 30 min and 24 h following exposure with NE, and/or Meth, with or without NAC or Cat. (C) Superoxide (O_2−_) detection was performed in BA cell cultures by staining with DHE. Representative images show a comparison of DHE intensity and pattern between BA and macrophages in culture, 30 min after exposure with NE or Meth, with or without NAC. (D) Normalized DHE fluorescence intensity, performed in Fiji/ImageJ. Two-way ANOVA was followed by Bonferroni’s *post hoc* test. *p < 0.05 in comparison to controls in each time point. # *p* < 0.05 in comparison to Meth group.

BA primary cultures and macrophages were isolated and tested regarding effects on the production of ROS, previously suggested as critical in BAT thermogenesis [6,7], as single-cell and co-cultures. The ability to produce ROS was estimated by the release of H_2_O_2_ in the culture supernatants both at thirty minutes, and 24 h after the stimulus, and by the detection of intracellular superoxide (O_2−_). We found that BA and macrophages differed in their ROS production in response to Meth and NE (Figure 1), in a time-dependent manner. At 30 min, both BA and macrophages responded to Meth with a significant increase in H_2_O_2_ release (Figure 1(A,B)), but only macrophages responded to NE (Figure 1(B)). Moreover, in BA Meth + NE showed H_2_O_2_ levels in the supernatant that was similar to controls (Figure 1(A)), while in macrophages H_2_O_2_ release in Meth + NE was significantly elevated in comparison to controls. At 30 min, intracellular O_2−_, detectable using the sensitive dye DHE and fluorescence microscopy, was increased by Meth in both cell types (Figure 1(C,D)). NAC and Catalase decreased O_2−_ levels and abrogated the release of H_2_O_2_ in both cell types. At 24 h, BA showed increased H_2_O_2_ release in culture supernatants stimulated with NE, but not with Meth (Figure 1(A)), while macrophage cultures had H_2_O_2_ levels higher than controls in response to Meth-stimulation, but not to NE (Figure 1(B)). In both cell types, levels of H_2_O_2_ following the treatment with the combination of Meth and NE were higher than controls (Figure 1(A,B)). In both cell types, NAC and Catalase had similar effects in maintaining H_2_O_2_ at control levels or below. This result indicates that both cell types may contribute to the oxidative stress response in BAT following Meth exposure, with potential consequences to the development of a thermogenic program. We examined the consequences of that exposure to mitochondrial health at 24 h after the exposure to Meth, NE or both.

**Figure 2.**
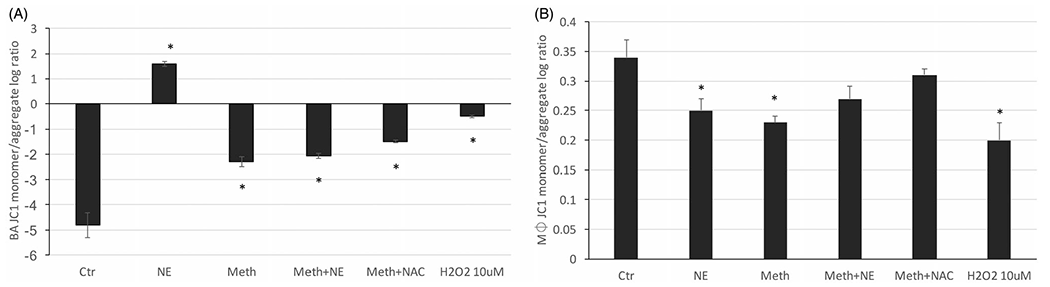
Changes in mitochondrial membrane potential induced by Meth and NE in BA and macrophages. JC1 was measured as the ratio between monomers and aggregates (A) in BA cultures, and (B) in macrophages, 24 h after treatment with vehicle, Meth, NE, with or without NAC. H_2_O_2_ was added as a control. ANOVA followed by Bonferroni’s *post hoc* test. *p < 0.05, indicates significant comparisons against vehicle-treated control cultures.

Mitochondrial function is linked to ROS production [32]. We have previously described that BA mitochondria is deeply affected by Meth *in vivo,* based on morphology and upregulation of mitochondrial complex I, detectable 24 h after drug injection [7]. We have also shown that BAT denervation prevented mitochondrial changes, suggesting that they may be largely mediated by sympathetic input in BAT, and in correlation with thermogenesis [7,33,34]. Here tested the role of NE and Meth on the physiological response of mitochondria *in vitro,* in isolated BA and macrophages. Mitochondrial health was estimated through measuring mitochondrial membrane potential using JC-1, following 24 h of stimulation. Our hypothesis posited that changes in membrane potential may be lined to, or result from, H_2_O_2_ release in a cell subset-specific manner. In order to test that, NAC was added as a scavenger of H_2_O_2_ along with Meth as a stimulant, while the addition of exogenous H_2_O_2_ was used to estimate the contribution of ROS to changes in mitochondrial health. The potential-dependent accumulation of JC-1 aggregates in mitochondria was indicated by a shift from green fluorescence emission (~529 nm) to red (~590 nm), while mitochondrial depolarization was revealed by an increase in the green/red intensity ratio, indicative of cellular stress. Interestingly, BA had a drastically lower baseline green/red (monomer/aggregates) ratio compared to macrophages (Figure 2), indicating intrinsic differences in baseline stress between the two cell types, which may account to a stronger response in less stressed BA, compared to macrophages. The stimulation with both Meth and NE alone significantly increased mitochondrial polarization in BA compared to vehicle. Moreover, the effect of NE alone was 50% stronger than the effect of Meth. The co-stimulation with Meth and NE caused the polarization to increase to levels that were similar to Meth alone, indicating an attenuating effect of NE. The addition of NAC to Meth had a modest effect, indicating that the Meth/NE-induced mitochondrial polarization in BA is only partially dependent on H_2_O_2_ (Figure 2). In macrophages, on the other hand, with mitochondria that are polarized at baseline, NE, Meth and H_2_O_2_ had modest effects on polarization, compared to the effects on BA. Yet, the effects on macrophages were statistically significant (Figure 2). The addition of NAC of Meth on macrophages prevented the increase in polarization, indicating a role for ROS in Meth-induced mitochondrial polarization in those cells.

**Figure 3.**
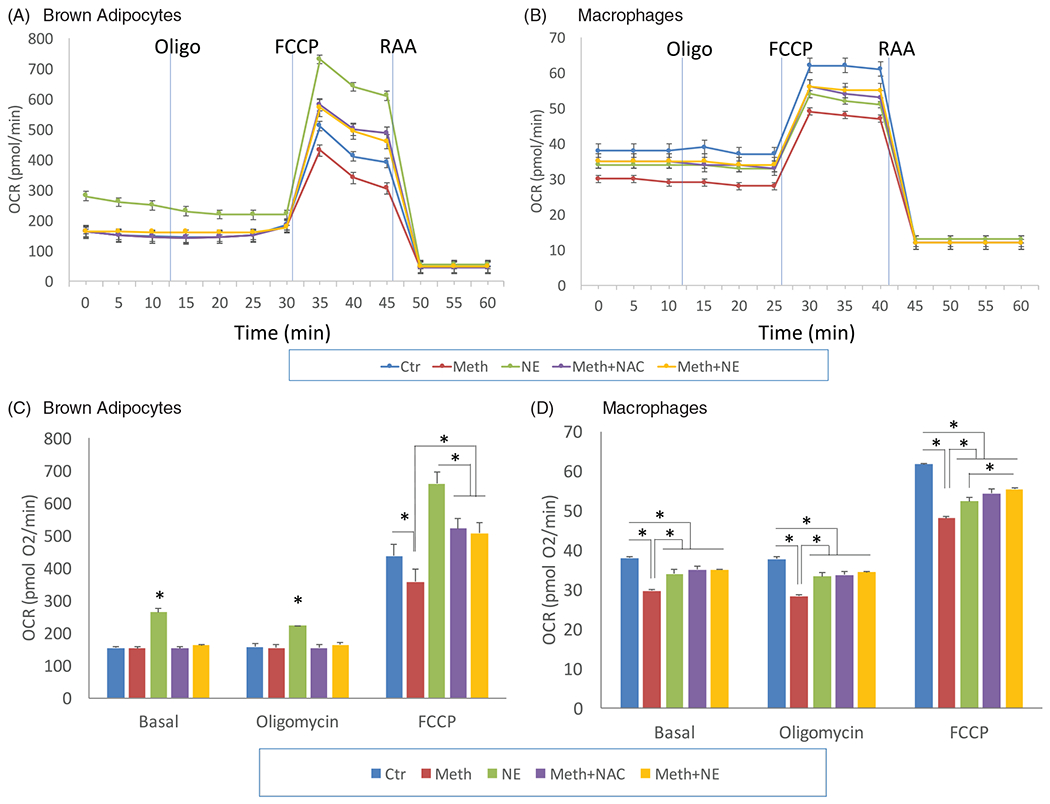
Oxygen consumption rate (OCR) in Meth and NE stimulation in BA and macrophages. OCR was recorded as an average of 3–4 wells containing 10^5^ cells. Media, Oligomycin (Oligo), FCCP, Rotenone and Antimycin A (RAA) were added as indicated. (A) BA and (B) Peritoneal macrophages were stimulated with 0.1 μM NE, 60 μM Meth or both. The contribution of ROS to the effects of Meth was examined by the addition of 5 pg/ml NAC, simultaneously with Meth. Bar graphs show the total OCR during basal, Oligomycin and FCCP stimulation in (C) BA and (D) macrophages. The experiment was replicated 3 times. *p < 0.05, ANOVA followed by Bonferroni’s test.

Another measure of mitochondrial activity is oxygen (O_2−_) consumption, largely through the mitochondrial respiratory chain, and which is a source of ROS production in reduced redox states [35], and an estimate of metabolic demands, including of thermogenesis. Mitochondrial oxygen consumption rate (OCR) was measured in BA and macrophages stimulated with NE and Meth, as indicative of changes in mitochondrial function (Figure 3), and validated by a Mito Stress test, consisting of the addition of an uncoupling agent FCCP, which disrupts adenosine triphosphate (ATP) synthesis by transporting hydrogen across the mitochondrial membrane and causing a rapid consumption of energy and oxygen. These measures were used to calculate the spare respiratory capacity in BA and in macrophages following NE and Meth. In BA, we found that Meth significantly decreased the maximal mitochondrial capacity by 10%, while NE improved it (Figure 3(A)). NAC prevented the effect of Meth, suggesting that the decrease in mitochondrial capacity caused by the drug may be associated with ROS production (Figure 3(A)). In macrophages, on the other hand, both Meth and NE decreased mitochondrial capacity by 15% and 10%, respectively (Figure 3(B)). Moreover, neither NAC or NE was able to significantly modify the effect of Meth in macrophage OCR, suggesting that ROS and sympathetic input do not contribute to mitochondrial respiratory activity in macrophages following Meth or NE exposure.

In summary, the two major cell types in BAT, BA and macrophages, are both affected by factors that are present following Meth exposure, including the drug itself and NE, which is delivered into the tissue through innervation and contributes to thermogenesis [6,7,23]. Both cell types differ at baseline and in response to Meth and NE, regarding ROS production, and mitochondrial functional changes.

Next, we examined whether NE and Meth modify cell-specific functions. In macrophages, we measured the oxidative stress response and cytokine transcriptional patterns. In BA, we measured changes in the transcription of genes involved in thermogenesis. Following that, we examined whether and macrophages are able to modulate the expression of the BA thermogenic program in co-cultures, stimulated with NE and Meth.

### Effects of meth and NE on oxidative stress and cytokine patterns in macrophages

**Figure 4.**
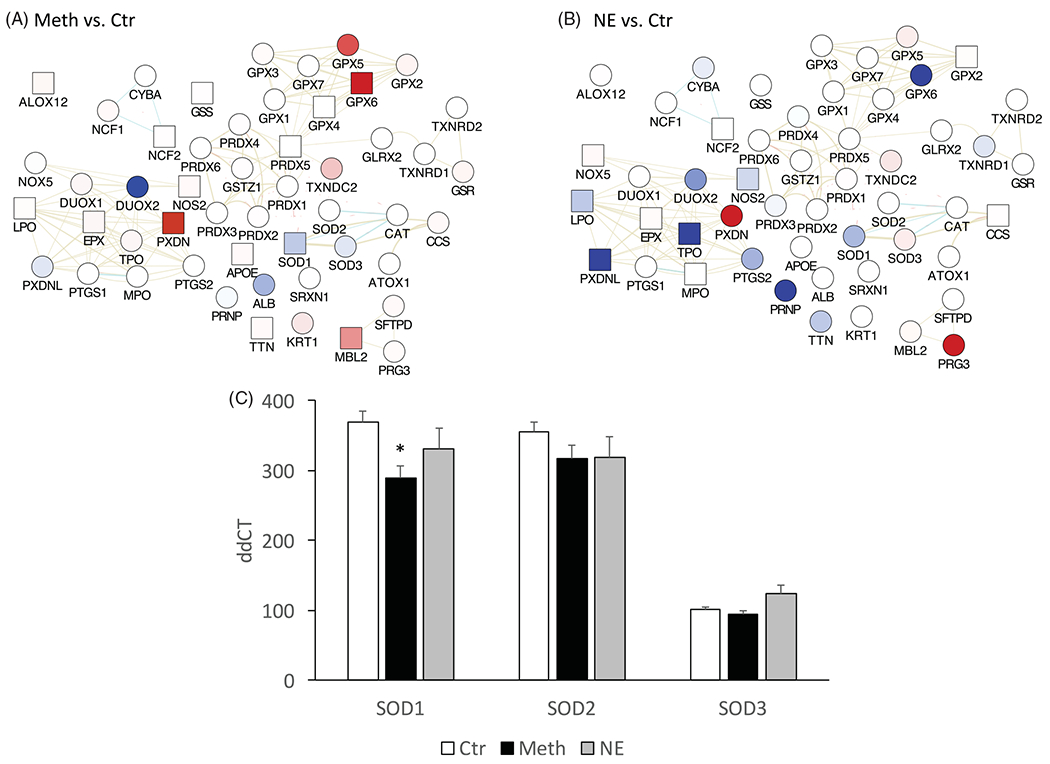
Oxidative stress gene expression in macrophages stimulated with Meth and NE in culture. Macrophages isolated from BAT were stimulated for 24 h with (A) Meth (60 μM) or (B) NE (0.1 μM), and the transcription of genes associated with oxidative stress was measured in targeted PCR arrays, with normalization performed using GAPDH and 18S. The functional interactions between the measured genes were visualized using Cytoscape, where blue shades in nodes represent downregulation, and red represents upregulation, when compared to unstimulated controls. Square nodes represent significance at *p* < 0.05 in each assigned comparison. (C) The transcriptional levels of superoxide dismutases 1, 2, and 3 (SOD1, SOD2 and SOD3), can be appreciated in isolation. Two-way ANOVA was followed by Bonferroni’s *post hoc* test. *p < 0.05 in comparison to the control group.

Macrophages isolated from BAT were stimulated with Meth or NE *in vitro* for 24 h, and the transcription of genes in the oxidative stress response was measured using pathway-targeted PCR arrays. As expected, both Meth and NE disturbed the oxidative stress response pathway, with common and distinctive effects (Figure 4). The transcription of the superoxide dismutase 1 (SOD1) was significantly decreased by Meth (Figure 4(A,C)), but only marginally by NE (Figure 4(B,C)). SOD2 showed a trend for downregulation when Meth or NE was added to macrophages, but this was not statistically significant, compared to vehicle-stimulation. SOD3 has shown a trend for downregulation with Meth and upregulation with NE, but these changes were not statistically significant. Other genes in the oxidative stress pathway that were significantly affected by Meth (Figure 4(A)), include the upregulation of Nitric Oxide Synthase 2 (NOS2, 1.51-fold, *p* = 0.001), Apolipoprotein E (APOE, 1.51-fold, *p* = 0.04), Arachidonate 12-Lipoxygenase (ALOX12, 1.5-fold, *p* = 0.01), Mannose-binding lectin 2 (MBL2, 5-fold, *p* = 0.04), glutathione peroxidase 6 (GPX6, 29-fold, *p* = 0.02), and peroxidasin (PXDN, 265-fold, *p* = 0.003). NE, on the other hand, mostly decreased the genes in this pathway in macrophages (Figure 4(B)), with significant effects in downregulating NOS2 (0.73-fold, *p* = 0.006), lactoperoxidase (LPO, 0.7-fold, *p* = 0.007), thyroid peroxidase (TPO, 0.38-fold, *p* = 0.009) and the peroxidasin-like precursor protein (PXDNL, 0.33-fold, *p* = 0.001). Similar to Meth, NE increased PXDN by 90-fold (*p* < 0.0001).

**Figure 5.**
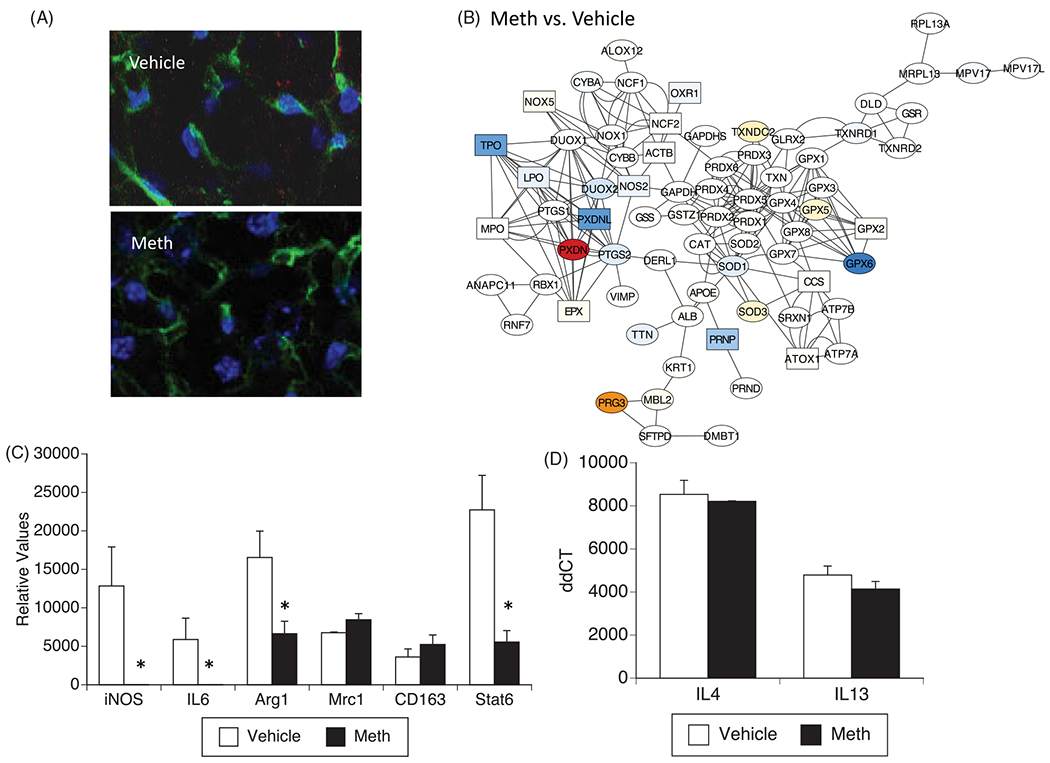
Phenotypes of macrophages isolated from BAT after vehicle or Meth injection. Macrophages were isolated from BAT 24 h after *in vivo* treatment with vehicle or 3 mg/kg of Meth ip. (A) The transcription of genes associated with oxidative stress was measured in targeted PCR arrays, with normalization performed using GAPDH and 18S. The functional interactions between the measured genes were visualized using Cytoscape, where blue shades in nodes represent downregulation, and red shades represent upregulation when compared to macrophages from vehicle-injected animals. Square nodes represent significance at *p* < 0.05 in each assigned comparison. Changes suggest similarities with patterns produced *in vitro* by NE treatment. (C) Transcriptional expression of genes that indicate macrophage subsets M1 (iNOS, IL6) and M2 (Arg1, Mrc1, CD163 and Stat6). (D) Transcriptional expression of IL4 and IL13 in BAT. These cytokines can increase and signal M2 macrophages to control thermogenic commitment in adipocytes [36]. The transcriptional changes were performed 24 h after treatment and were examined by SyBrGreen qRT-PCR. Relative values were normalized based on GAPDH. Values represent the average ± SD of four replicates/group. *p < 0.05 Compared to respective controls.

Following this *in vitro* characterization, we performed the characterization of macrophages isolated from BAT, 24 h after Meth injection, and compared to macrophages from animals stimulated with vehicle (Figure 5). Morphologically, the macrophages in BAT did not differ significantly between Meth and vehicle stimulation (Figure 5(A)). However, compared to the vehicle, they had a decreased expression of many genes in the oxidative stress pathway, in a pattern that closely resembled the *in vitro* stimulation with NE (Figure 5(B)). Significantly decreased genes in that pathway included oxidation resistant-1 (OXR1, 0.83-fold, *p* = 0.05), NOS2 (0.62-fold, *p* = 0.03), LPO (0.68-fold, *p* = 0.01), prion protein (PRNP, 0.63-fold, *p* = 0.05), TPO (0.25-fold, *p* = 0.01), and PXDNL (0.25-fold, *p* = 0.01). Oxidative stress genes that were significantly upregulated included the copper metallochaperone antioxidant 1 (ATOX1, 1.22-fold, 0.05), glutathione peroxidase 2 (GPX2, 1.44-fold, *p* = 0.009), the calcium-dependent NADPH oxidase 5 (NOX5, 1.23-fold, *p* = 0.006), the neutrophil cytosolic protein 2 (NCF2, 1.26-fold, *p* = 0.0009, a calcium-dependent subunit of the NADPH-oxidase complex) and the eosinophil peroxidase (EPX, 1.34-fold, *p* = 0.02). Moreover, BAT-derived macrophages abrogated the expression of genes associated with the M1 subset, iNOS and IL6 by qRT-PCR, at 24 h post-Meth, compared vehicle (Figure 5(C)). The expression of Arg1 and Stat6, which are genes that characterize the M2 subset, was significantly decreased, but not abolished by Meth *in vivo,* indicating a predominance of M2 subsets in BAT following Meth (Figure 5(C)). Meth treatment did not change the expression of cytokines IL13 and IL4 in BAT-derived macrophages (Figure 5(D)). These cytokines are involved in M2 polarization [37] and have been described to influence thermogenesis in models of cold exposure, causing myeloid production of catecholamines [9]. However, Meth does not induce catecholamine production in macrophages [12].

In summary, macrophages from BAT are influenced both by Meth directly and by NE. Transcriptionally, genes in the oxidative stress pathway expressed by macrophages isolated from BAT 24 h after Meth resemble patterns that are induced by NE *in vitro.* Moreover, Meth induces a down-modulation of macrophage subset markers and a predominance of cells with an M2 phenotype.

### Effects of meth and NE on BA genes associated with the thermogenic program in BA

**Figure 6.**
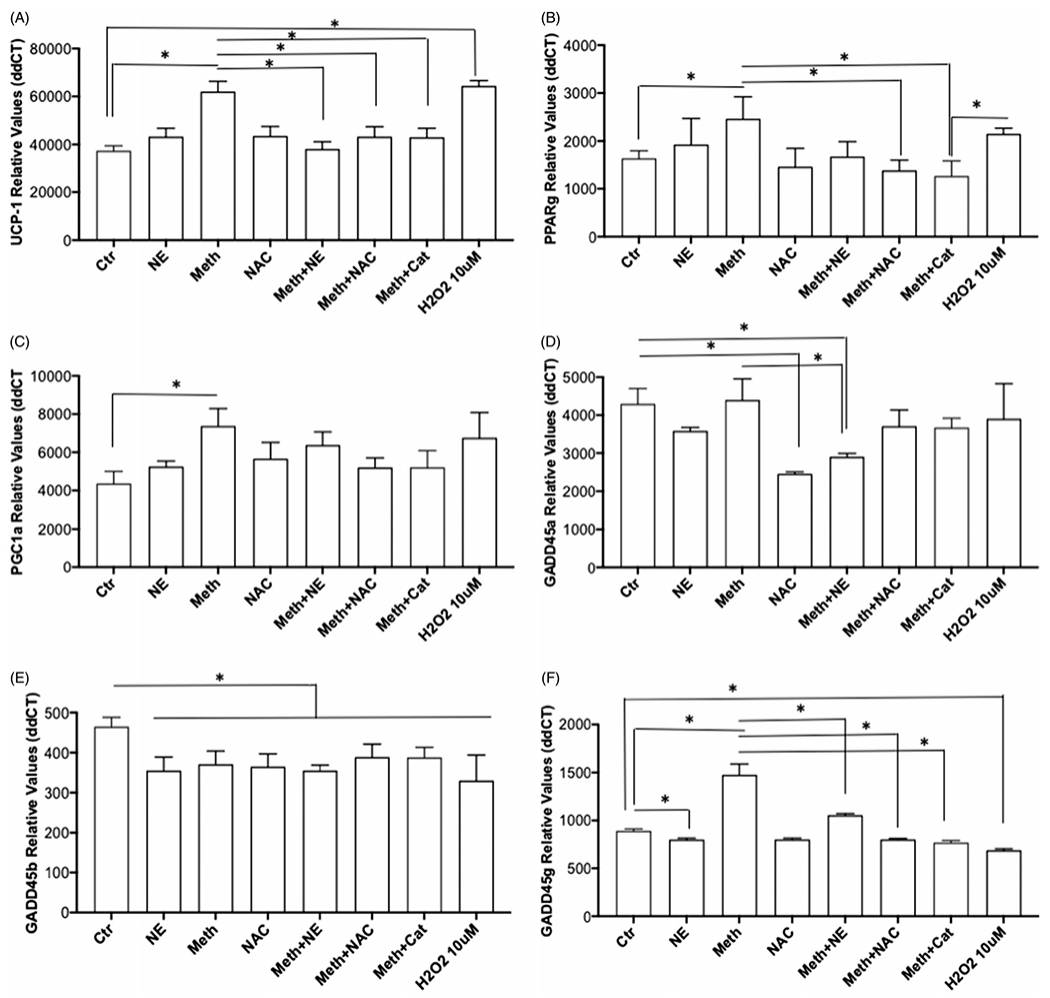
Effect of Meth and NE, and contribution of ROS, on the expression of genes associated with and regulating thermogenesis in BA. Transcriptional levels of (A) UCP1, (B) PPARγ, (C) PGC1α, (D) GADD45α, (E) GADD45β and (F) GADD45γ were measured in primary cultures at 24 h after stimulation with 0.1 μM NE, 60 μM Meth or both, 5 pg/ml NAC, or 2 U/ml of Catalase (Cat) using SyBrGreen qRT-PCR. Relative values were normalized based on GAPDH. Values represent the average ± SD of 8 technical replicates/group in four experiments. *p < 0.05 compared to respective controls.

Changes in the transcription of genes that play important roles in thermogenesis within BAT may indicate how the influence of Meth on BA occurs, and whether this is through NE or ROS. Among thermogenesis-relevant genes in BAT, UCP1 is critical in the generation of heat from mitochondrial respiration [38]. In BA primary cultures, UCP1 transcription was modulated by treatment, addressing the effects of Meth, NE, and ROS (ANOVA, *p* < 0.0001). In multiple comparisons, we found that Meth, but not NE, significantly increased UCP1 (Figure 6(A), *p* = 0.0007). The expression of UCP1 in BA cells treated with Meth together with NE (Meth + NE), with NAC (Meth + NAC), or with Catalase (Meth + Cat) was significantly lower than with Meth alone (*p* = 0.0011, *p* = 0.0187 and *p* = 0.0162, respectively). Interestingly, the exposure of BA cells to H_2_O_2_ elevated UCP1 expression (*p* = 0.0001). These results suggest that ROS is important in the upregulation of UCP1 by Meth in BA.

Both PPARγ and PGC1α regulate the expression of UCP1 [39]. There was an effect of treatment on the BA transcription of PPARγ (Figure 6(B), ANOVA *p* < 0.0001). This effect was characterized by an increase induced by Meth (*p* = 0.0325), which was prevented in the presence of NAC (*p* = 0.0023), as well as of Catalase (*p* = 0.0006), but not significantly by NE (*p* = 0.056). PGC1α transcription also showed an effect of treatment (Figure 6(C), ANOVA *p* = 0.0004). The effect on PGC1a was characterized by a significant increase in its transcription by Meth (*p* = 0.0004), which was prevented by NAC (*p* = 0.0161), and by Catalase (*p* = 0.0172), but not NE (*p* = 0.6963). These results suggest that ROS play a role in the increase of PPARγ and PGC1α induced by Meth in BA.

We also examined the effect of treatment on the expression of other important genes involved in the regulation of thermogenic and stress-response pathways. These genes included GADD45α, β, and γ (Figure 6(D–F), respectively). Regarding GADD45α, we have detected an effect of treatment (Figure 6(D), ANOVA *p* < 0.0001), which was characterized by a significant decrease induced by Meth + NE, and by NAC alone, when compared to controls (*p* < 0.0001, and *p* = 0.0007, respectively). Meth + NAC was also significantly lower when compared to Meth alone (*p* = 0.0003). This suggests that ROS induced by Meth does not affect the expression of GADD45α, but NE may be a factor in its regulation. The expression of GADD45β was affected by treatment (Figure 6(D), ANOVA *p* < 0.0001). In multiple comparisons, this effect was characterized by a significant decrease in the transcription of the gene, caused by Meth (*p* = 0.0058), by NE (*p* = 0.0008), and by the combination of Meth + NE (*p* = 0.0008). NAC, Meth + NAC and Meth + Cat, also caused a significant decrease in GADD45β compared to control conditions (*p* = 0.0027, *p* = 0.046 and *p* = 0.0401, respectively). Interestingly, H_2_O_2_ significantly decreased the expression of GADD45β as well (*p*< 0.0001), indicating that ROS alone does not explain these changes. Together, the results suggest that the expression of GADD45β is sensitive to a diversity of stimuli causing its downregulation, including, but not restricted to, ROS and NE. The effects of treatment on the transcription of GADD45γ (Figure 6(F), ANOVA *p* < 0.0001), was also characterized by a significant increase caused by Meth (*p* < 0.0001), and by Meth + NE (*p* < 0.0001). However, the expression of GADD45γ in BA cells stimulated with Meth + NE was significantly lower than with meth alone (*p* < 0.0001). NE caused a decrease in GADD45γ expression compared to control (*p* = 0.0184). Importantly, both NAC and Catalase prevented the increase in GADD45γ induced by Meth (*p* < 0.0001, and *p* < 0.0001, respectively). Yet, H_2_O_2_ alone had no effect. This suggests that ROS alone is not enough for enhancing downstream thermogenic pathways that are triggered by Meth.

**Figure 7.**
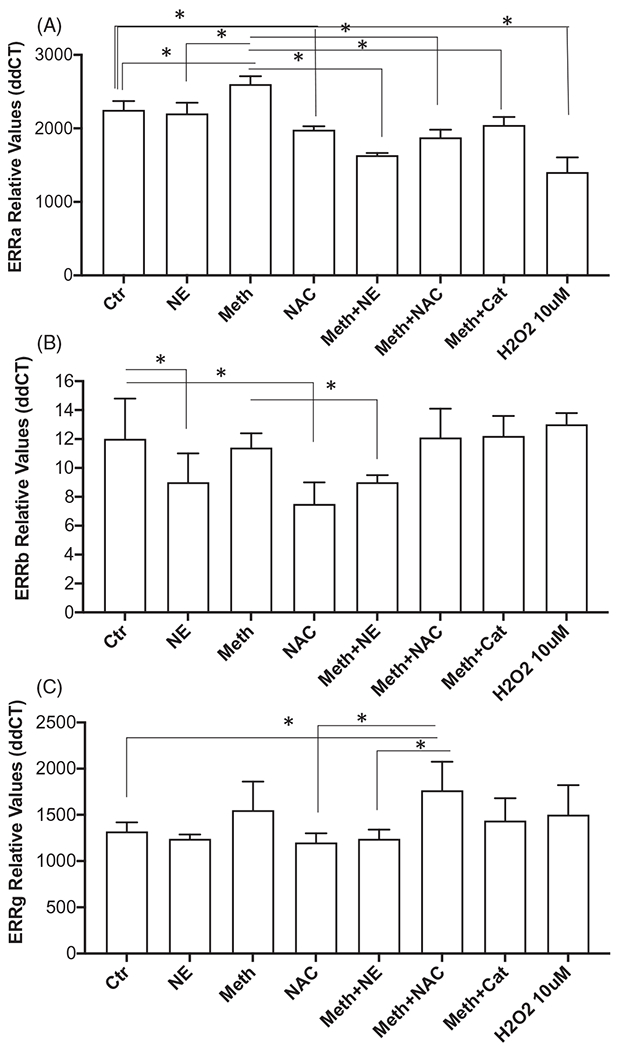
Effect of Meth and NE, and contribution of ROS, on the expression of signaling components of the ERR gene family in BA primary cultures. Transcriptional levels of (A) ERRα, (B) ERRβ, and (C) ERRγ were measured at 24 h after stimulation with 0.1 μM NE, 60 μM Meth or both, 5 pg/ml NAC, or 2 U/ml of Cat. using SyBrGreen qRT-PCR. Relative values were normalized based on GAPDH. Values represent the average ± SD of 8 technical replicates/groups in four experiments. *p < 0.05 compared to respective controls.

We examined the impact of Meth on the transcription of signaling components of the GADD45 pathway, ERRα, ERRβ and ERRγ. The Estrogen-related receptors (ERR) family has been shown to be critical as adrenergic effectors in adaptive thermogenesis in BAT [40]. We tested their expression in cultured BA stimulated for 24 h with Meth, NE, and both, and we addressed the role of ROS in changes caused by Meth (Figure 7(A–C)). There was an effect of treatment on the expression of ERRα (Figure 7(A), ANOVA *p* < 0.0001). In multiple comparisons, we found that the effect was characterized by a significant increase on ERRα expression caused by Meth in comparison to Ctr (*p* < 0.0001). NE alone did not affect ERRα significantly, but Meth + NE decreased the expression of the gene compared to Ctr and to Meth alone (*p* < 0.0001 and *p* < 0.0001). Both Meth + NAC and Meth + Cat decreased ERRα compared to Meth alone (*p* < 0.0001 and *p* < 0.0001). However, H_2_O_2_ had a suppressive effect on the expression of the gene compared to Ctr. Regarding the expression of ERRβ, there was an effect of treatment (Figure 7(B), ANOVA, *p* < 0.0001), characterized by a significant decrease caused by NE (*p* = 0.016), and by NAC (*p* < 0.0001), in comparison to Ctr. Meth alone did not affect the expression of ERRβ, alone or together with NAC (*p* = 0.998 and *p* = 0.945). H_2_O_2_ also did not affect the expression of this gene (*p* > 0.999). Yet, Meth + NE caused a decrease in ERRb expression, to levels similar to NE alone (*p* = 0.017). This suggests that transcription of ERRβ may be predominantly affected by NE. The expression of ERRγ was affected by treatment (Figure 7(C), ANOVA, *p* < 0.0001). Multiple comparisons have shown that although Meth and NE, alone or combined, did not cause significant changes in the expression of this gene, Meth + NAC significantly increased the expression of ERRγ in comparison to Ctr (*p* = 0.0075). Yet, the expression in Meth + NAC-treated cells did not differ from Meth alone. Other treatments did not affect the expression of ERRγ significantly.

Overall, the transcriptional expression of genes that participate in the BA thermogenic program is upregulated by a combination of effects resulting from NE and of factors associated with the drug, including, but not restricted to ROS.

### Effects of macrophages on the changes in the BA thermogenic program caused by meth and NE

We compared primary BA cultures in the presence and absence of macrophages, in order to test their contribution to changes in the transcriptional phenotypes in genes associated with the thermogenic program in BAT, and that are caused by Meth or by NE.

**Figure 8.**
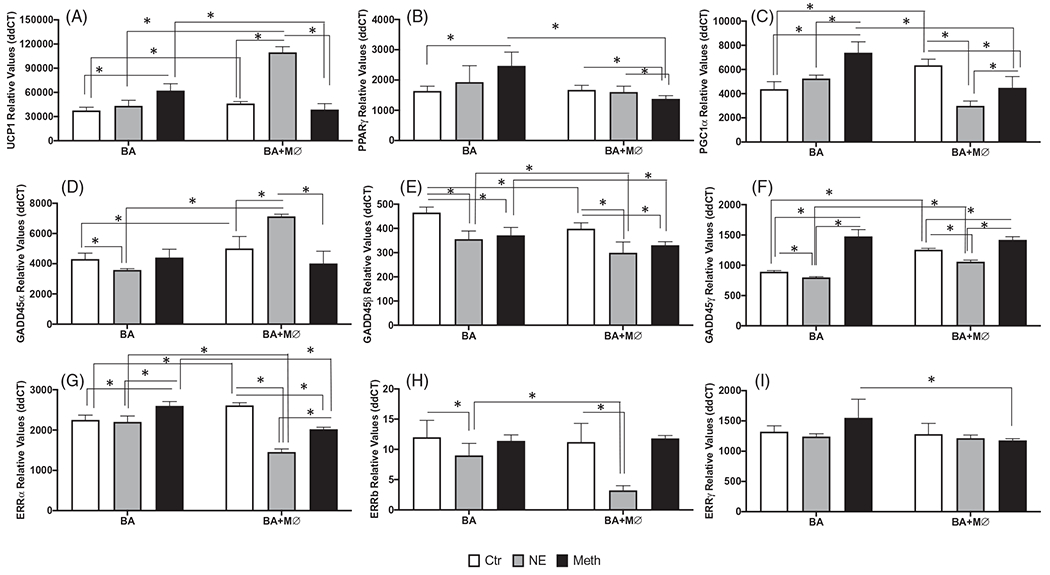
Effects of macrophages co-cultured with BA on transcriptional levels of thermogenic genes, after stimulation with Meth or NE. Transcriptional levels of (A) UCP1, (B) PPARγ, (C) PGC1α, (D) GADD45α, (E) GADD45β, (F) GaDD45γ, (G) ERRα, (H) ERRβ and (I) ERRγ were measured at 24 h after stimulation with 0.1 μM NE, or 60 μM Meth by SyBrGreen qRT-PCR. Relative values were normalized based on GAPDH. Values represent the average ± SD of 6–12 technical replicates/groups in four independent experiments. *p < 0.05 in indicated comparisons.

While in primary BA cultures, Meth upregulated UCP1, PPARγ and PGC1α (Figure 6(A–C)), in the presence of macrophages these genes were decreased in comparison to vehicle control (Figure 8(A–C)). For instance, in co-cultures, the expression of both UCP1 and PGC1a was modestly but significantly increased in unstimulated control conditions in comparison to BA cells alone (Figure 8(A,C), *p* = 0.005, and *p* < 0.0001, respectively). Meth stimulation did not increase the transcription of UCP1 significantly in co-cultures, while NE caused a significant increase both in comparison to control co-cultures, and to Meth stimulation in BA alone (Figure 8(A), both *p* < 0.0001). Regarding PPARγ expression, we found that while Meth caused a significant increase in BA single cultures, the transcription of this gene was maintained at low levels in co-cultures (Figure 8(B), *p* < 0.0001 Meth in BA + MØ in comparison to BA alone). In single BA cultures, Meth also increased PGC1α compared to control (Figure 8(C), *p* < 0.0001), while in the presence of macrophages the expression of PGC1α was decreased, both with Meth and with NE, in comparison to stimulation in BA alone (*p* < 0.0001). These results indicate that macrophages largely regulate BA response to Meth, and to NE, and elevated the baseline threshold transcription.

The stress-responsive thermogenic genes in the GADD45-pathway were also impacted differently by Meth and NE in the presence of macrophages. For instance, the expression of GADD45α, was downregulated by NE in BA alone (*p* = 0.009), and not affected by Meth stimulation (Figure 8(D)). However, in co-cultures, GADD45α was significantly upregulated by NE (*p* < 0.0001). The expression of GADD45β (Figure 8(E)) was also modified by macrophages in co-culture, which caused a decrease in the expression of those genes in baseline conditions and upon stimulation with NE or Meth (*p* < 0.0001). However, we did not observe any effect of interaction between treatments and macrophages in the expression of this gene (*p* = 0.546). The transcriptional expression of GADD45γ in both unstimulated controls and NE-stimulated cells was increased in the presence of macrophages (*p* < 0.0001), compared to BA alone (Figure 8(F)). When Meth was the stimulus, GADD45γ expression was increased in comparison to controls, in both BA alone and in BA + MØ (*p* < 0.0001).

In co-cultures, the expression of ERRα in unstimulated controls was significantly higher than in BA alone (*p* < 0.0001). While in BA cells alone NE did not affect the expression of ERRα, it decreased its expression in co-cultures (*p* < 0.0001, Figure 8(G)). Meth, on the other hand, increased ERRα in BA cultures but decreased the expression of this gene in BA + MØ (*p* < 0.0001, Figure 8(G)). The expression of ERRβ (Figure 8(H)) was decreased by NE both in BA single cultures (*p* = 0.0056) and in co-cultures with macrophages (*p* < 0.0001). Moreover, this NE-induced decrease was more prominent in co-cultures compared to BA alone (*p* < 0.0001). Regarding ERRγ, although there was no effect of treatment, a modest but significant impact of macrophages in co-culture (2-Way ANOVA, *p* = 0.021) was observed (Figure 8(I)). This impact was due to a lower expression of ERRγ in BA + MØ stimulated with Meth, compared to Meth stimulation in BA cultures (*p* = 0.0063).

Overall, the results suggest that macrophages have a strong impact on the transcription of genes involved in BAT thermogenesis in the context of Meth and upon local input of sympathetic responses. Our results show that macrophages largely modulate the response to NE and to Meth, with implications for the development of a transcriptional thermogenic program.

## Discussion

Recent studies have identified active BAT in human adults [41]. BAT is a tissue with a known role in thermogenesis [42], and which we have shown to play an important role in the life-threatening phenomenon of hyperthermia, induced by psychostimulants such as Meth [6,7,23]. Here we have characterized the impact of Meth on the two major cell types represented in the BAT, macrophages and BA. In addition to the effects of direct Meth exposure, we also tested these cells’ responses to NE, which we have previously demonstrated to contribute to hyperthermia *in vivo* [7]. With that, we used *in vitro* primary culture and co-culture approaches to investigate the response of macrophages and BA, and examined whether the development of a molecular thermogenic program triggered in the context of Meth is impacted by the interaction between these two cell types. We examined the hypothesis that changes in ROS levels in response to Meth, directly or through NE, may be associated with the development of a thermogenic environment. We have shown that both BA and macrophages are responsive to NE and Meth with the production of O_2−_ and release of H_2_O_2_, in a time-sensitive manner. NAC, as well as Catalase, which significantly decreased or abrogated the synthesis of ROS in both cell types, modified the Meth-induced transcription of thermogenesis-relevant genes in BA, including UCP1, PPARγ, PGC1α, and GADD45γ. These findings in culture further suggest a beneficial effect of anti-oxidants in drug-induced hyperthermia and a mechanism for that effect.

Our previous *in vivo* observations [7] have suggested that mitochondria and mitochondrial functions are deeply impacted by Meth, both directly and through NE. Yet, oxygen consumption experiments showed that in BA, Meth exposure decreased mitochondrial respiratory spare capacity, in a ROS-dependent manner, while NE caused spare capacity to be improved. It has been reported that a decrease in spare capacity is a consequence of aging [43], and suggesting a potential mechanism by which Meth may accelerate the aging process, and promote cytotoxicity [44]. Yet, Meth did not affect the release of H_2_O_2_ from BA, while NE caused a significant increase in culture supernatants. Our previous studies have shown that *in vivo*, BA in BAT do increase ROS following Meth injection, which is characterized by mitochondrial O_2−_ and H_2_O_2_ [23], and followed by mitochondrial damage [7]. Our *in vitro* results suggest that NE is a key factor for these effects in BA *in vivo*. In macrophages, on the other hand, both Meth and NE decreased spare capacity, but H_2_O_2_ was rather induced by Meth. In macrophages, ROS are important in defense, and the majority of ROS are derived from NADPH oxidases (NOX). It has been suggested that the exposure of macrophages to Meth can activate the NOX complex toward ROS production [45]. Our results suggest a contribution of both BA and macrophages to the redox environment in BAT after Meth stimulation, through distinct cell-specific mechanisms.

The examination of transcriptional changes in BA and macrophages was targeted to genes that have been described as relevant to thermogenesis in this and other models. The treatment of cells with the ROS scavenger NAC prior to Meth was performed to test the impact of ROS as a signaling element in thermogenesis. We previously showed that NAC can prevent the onset of hyperthermia and also rescue animals after the Meth-hyperthermia process was initiated [6]. NAC may act as a reduced glutathione (GSH) precursor, but its action can also be through its thiol-disulfide exchange activity [46,47], which can cause proteins to change structure and function. Catalase as a control might act redundantly when the effects on transcription are associate with ROS, but could differ if the effects of NAC are associated with the reduction of cysteine thiols in proteins that are relevant to thermogenesis.

We have shown that ROS is involved in modulating Meth thermogenesis *in vivo* [6,7]. We found that *in vitro,* Meth downregulated SOD1 in macrophages, and increased a network of genes involved in oxidative stress response. NE, on the other hand, downregulated several genes in that pathway. Macrophages isolated from BAT after a Meth injection showed a transcriptional profile that was similar to what was induced by NE in the *in vitro* assays, rather than by Meth. *In vivo,* we have previously shown that hyperthermia is significantly less pronounced in animals with denervated BAT, in which the influx of sympathetic mediators has been surgically interrupted [7]. Given the effect of NE on macrophages, and the effect of macrophages on BA thermogenic program, these observations support a cross-communication between these cell types, both with indirect and direct effects. BA produced higher amounts of ROS compared to macrophages, which account for mitochondrial changes and contribute to signaling thermogenesis [1,2,4], driven by high oxygen consumption [48]. Meth-induced hyperthermia is followed by morphological changes in BAT mitochondria suggestive of activation and damage, and mainly as a result of post-ganglionic sympathetic neuron release of NE [7,49]. Likewise, others have shown that the thermogenic capacity of BAT as an adaptive response to cold under control of NE is dependent on the activation of a thermogenic program that includes the uncoupling protein 1 (UCP1), PPARγ and PGC1α, and downstream signaling in BA [33,50]. BAT mitochondria have an intrinsically highly oxidized status and high basal ROS emission compared to other mitochondria-rich sites, such as skeletal muscle [48]. A reason for that is the low expression of glutathione, and the absence of key redox enzymes in BAT tissue [48], which are critical characteristics for UCP1 activity in thermogenesis [48].

The transcription of UCP1, PPARγ, and PGC1α, was upregulated in BA cultures by Meth, but not by NE, and this upregulation was prevented with NAC and with Catalase. In the GADD45 pathway, only GADD45γ was increased by Meth, and controlled by Meth + NE, as well as by NAC. On the other hand, when BA and macrophages were co-cultured, NE enhanced the transcription of UCP1 and GADD45α, and the effects of Meth were largely attenuated in comparison to BA alone. This suggests that macrophages can play a critical role at maintaining homeostasis in peripheral thermogenic sites. The GADD45 pathway is induced by environmental stress and is associated with DNA damage and other stress signals associated with growth arrest and apoptosis [51]. Distinct roles are attributed to each one of the subunits [52]. GADD45α is an RNA-binding protein, which can inhibit autophagy [53,54]. GADD45β and GADD45γ specifically interact with the Cdk1/CyclinB1 complex, with consequences to the cell cycle [55]. GADD45γ does not disrupt this complex, but can regulate cell cycle as well as other functions at the epigenetic level, by interacting with DNA double-strand break (DSB)-mediated changes in DNA methylation, and influencing early gene expression [56]. This mechanism described in association with neuronal development and learning [56], maybe active in other cell types. The GADD45γ/p38/ERRγ pathway was shown to regulate BAT thermogenesis [57]. While mice that lack PGC1a have only mild thermogenesis effects, mice that lack GADD45γ have important defects in UCP1 induction and in the thermogenic response in models of cold exposure [57]. GADD45γ is transcriptionally elevated by Meth in BA, regardless of macrophages in culture. In addition, NAC in BA single cultures prevents the transcription of this gene, making GADD45γ a good candidate for regulation of Meth hyperthermia. Experiments using knock out animals are necessary to identify the precise regulator, and whether the changes observed in BA can result in the modulation of hyperthermia *in vivo.*

In the ERR family, Meth did not have a robust effect on transcription, but NE showed a regulatory capacity, confirming the sensitivity of this pathway to sympathetic stimulation [40]. GADD45γ has been implicated in BAT thermogenesis in models of cold exposure and adrenergic stimulation, as an activator of ERRβ and ERRγ via cAMP and PKA, in BA [57]. ERRβ and γ then act in the transcription of thermogenic genes [57,58]. We find that Meth plays a marginal role in enhancing the transcription of ERR family genes in BA cultures. In the presence of macrophages, ERR-family genes were downregulated, especially in NE-exposed co-cultures. These genes have been shown to be involved in sensitizing BAT to adrenergic signaling, which leads to an increase in energy expenditure [40,58]. On the other hand, the impact of Meth on the transcription of effector thermogenesis genes such as UCP1, PGC1γ and PPARγ was significant, and ROS-dependent.

Isolated BAT macrophages from Meth-stimulated animals show downregulation of M1 subset markers (IL6 and iNOS), with the maintenance of M2 markers. Similar phenotypic changes have been observed in BAT macrophages in models of cold exposure, in association with a capacity to produce catecholamines [9]. In that model, IL4 and IL13 were shown to be critical for sustaining adaptive thermogenesis [9]. Different from cold-exposure, we have shown that Meth does not cause macrophages to produce catecholamines [12]. Moreover, transcriptional levels of IL4 and IL13 were not affected by Meth exposure. However, we have not tested whether Meth-hyperthermia can develop in animals that lack IL4 and IL13 expression.

We have made several attempts to include NAC (and Catalase) in co-cultures, but the effect of these anti-oxidants in those conditions was similar to what we observed in single BA cultures and more importantly, they masked the effects of macrophages (not shown). Other strategies must be developed to sort the contribution of ROS originated from macrophages, or the contribution of ROS from BA in the presence of macrophages, to signaling thermogenic genes in BA.

The mechanisms by which macrophages modulate the expression of genes in BA remain to be examined. Moreover, it is important to acknowledge that our study is limited to the transcription of thermogenic genes, without addressing protein function. Further studies are necessary to measure protein levels and activity in the BA, in perspective with changes in the thermogenic pathway, and in CBT, in the context of Meth exposure.

Overall, our results suggest that following Meth exposure, a communication loop occurs between sympathetic signals and ROS in BA and macrophages in BAT, with consequences to CBT. The combination of ROS-dependent and independent factors, adrenergic input, and cellular interactions modify BA mitochondrial functions and gene transcription, with effects on the development of a thermogenic program with potentially lethal consequences. Macrophages and ROS may be considered as potential targets to control psychostimulant-induced hyperthermia.

## Figures and Tables

**Table 1.** Primers pairs used in this study, purchased from Qiagen.

Gene symbol	Gene name	Catalog number
*UCP1*	Uncoupling protein 1	PPM05164B
*PPARγ*	Peroxisome proliferator activated receptor gamma	PPM05108C
*PGC1α*	Peroxisome proliferator-activated receptor gamma, coactivator 1 alpha	PPM03360I
*SOD1*	Superoxide dismutase 1	PPM03582A
*GADD45α*	growth arrest and DNA-damage-inducible 45 alpha	PPM02927C
*GADD45β*	growth arrest and DNA-damage-inducible 45 beta	PPM03566A
*GADD45γ*	growth arrest and DNA-damage-inducible 45 gamma	PPM05000B
*ERRα*	Estrogen-related receptor alpha	PPM05772G
*ERRβ*	Estrogen-related receptor beta	PPM34303A
*ERRγ*	Estrogen-related receptor gamma	PPM30362A
*IL6*	Interleukin 6	PPM03015A
*CD163*	Scavenger receptor cysteine-rich type 1 protein M130	PPM06162B
*Mrc1*	mannose receptor, C type 1	PPM24735A
*Arg1*	Arginase-1	PPM31770C
*NOS2*	nitric oxide synthase 2, inducible	PPM02928B
*Stat6*	signal transducer and activator of transcription 6	PPM04646F
*IL4*	Interleukin 4	PPM03013F
*IL13*	Interleukin 13	PPM03021B
*GAPDH*	Glyceraldehyde 3-phosphate dehydrogenase	PPM02946E
*18S*	18S ribosomal RNA	PPM72041A

## References

[1] Alcala M, Calderon-Dominguez M, Bustos E, Increased inflammation, oxidative stress and mitochondrial respiration in brown adipose tissue from obese mice. Sci Rep. 2017;7(1):16082. 29167565 10.1038/s41598-017-16463-6 PMC5700117

[2] Alcala M, Calderon-Dominguez M, Serra D, Mechanisms of impaired brown adipose tissue recruitment in obesity. Front Physiol. 2019;10:94. 30814954 10.3389/fphys.2019.00094 PMC6381290

[3] Bowyer JF, Hanig JP. Amphetamine- and methamphetamine-induced hyperthermia: implications of the effects produced in brain vasculature and peripheral organs to forebrain neurotoxicity. Temperature. 2014;1(3):172–182. 10.4161/23328940.2014.982049 PMC5008711 27626044

[4] Calderon-Dominguez M, Alcala M, Sebastian D, Brown adipose tissue bioenergetics: a new methodological approach. Adv Sci. 2017;4(4):1600274. 10.1002/advs.201600274 PMC5396156 28435771

[5] Ud M, Raiko J, Saari T, Human brown adipose tissue [(15)O]O2 PET imaging in the presence and absence of cold stimulus. Eur J Nucl Med Mol Imaging. 2016;43(10):1878–1886. 26993316 10.1007/s00259-016-3364-y PMC4969352

[6] Sanchez-Alavez M, Bortell N, Galmozzi A, Reactive oxygen species scavenger N-acetyl cysteine reduces methamphetamine-induced hyperthermia without affecting motor activity in mice. Temperature. 2014;1(3):227–241. 10.4161/23328940.2014.984556 PMC4557806 26346736

[7] Sanchez-Alavez M, Conti B, Wood MR, Bortell N, ROS and sympathetically mediated mitochondria activation in brown adipose tissue contribute to methamphetamine-induced hyperthermia. Front Endocrinol. 2013;4:44. 10.3389/fendo.2013.00044 PMC3632801 23630518

[8] Wolf Y, Boura-Halfon S, Cortese N, Brown-adipose-tissue macrophages control tissue innervation and homeostatic energy expenditure. Nat Immunol. 2017;18(6):665–674. 28459435 10.1038/ni.3746 PMC5438596

[9] Nguyen KD, Qiu Y, Cui X, Alternatively activated macrophages produce catecholamines to sustain adaptive thermogenesis. Nature. 2011;480(7375):104–108. 22101429 10.1038/nature10653 PMC3371761

[10] Martinez FO, Gordon S. The M1 and M2 paradigm of macrophage activation: time for reassessment. F1000Prime Rep. 2014;6:13. 24669294 10.12703/P6-13 PMC3944738

[11] Yu Q, Zhang D, Walston M, Chronic methamphetamine exposure alters immune function in normal and retrovirus-infected mice. Int Immunopharmacol. 2002;2(7):951–962. 12188036 10.1016/s1567-5769(02)00047-4

[12] Basova L, Najera JA, Bortell N, Dopamine and its receptors play a role in the modulation of CCR5 expression in innate immune cells following exposure to methamphetamine: implications to HIV infection. PLoS One. 2018;13(6):e0199861. 29944719 10.1371/journal.pone.0199861 PMC6019408

[13] Negus SS, Mello NK, Blough BE, Monoamine releasers with varying selectivity for dopamine/norepinephrine versus serotonin release as candidate “agonist” medications for cocaine dependence: studies in assays of cocaine discrimination and cocaine self-administration in rhesus monkeys. J Pharmacol Exp Ther. 2007; 320(2):627–636. 17071819 10.1124/jpet.106.107383

[14] Rothman RB, Baumann MH. Monoamine transporters and psychostimulant drugs. Eur J Pharmacol. 2003;479(1–3):23–40. 14612135 10.1016/j.ejphar.2003.08.054

[15] Flaim KE, Horwitz BA, Horowitz JM. Coupling of signals to brown fat: alpha- and beta-adrenergic responses in intact rats. Am J Physiol. 1977;232(3):R101–R109. 842692 10.1152/ajpregu.1977.232.3.R101

[16] Herd PA, Horwitz BA, Smith RE. Norepinephrine-sensitive Na+− K + ATPase activity in brown adipose tissue. Experientia. 1970; 26(8):825–826. 4247576 10.1007/BF02114197

[17] Horwitz BA, Detrick JR, Smith RE. Norepinephrine-induced thermogenesis: effect of interscapular brown fat. Experientia. 1972;28(3):284–286. 5026433 10.1007/BF01928691

[18] Horwitz BA, Herd PA, Smith RE. Effect of norepinephrine and uncoupling agents on brown tissue. Can J Physiol Pharmacol. 1968;46(6):897–902. 4177323 10.1139/y68-139

[19] Horwitz BA, Horowitz JM Jr, Smith RE. Norepinephrine-induced depolarization of brown fat cells. Proc Natl Acad Sci USA. 1969; 64(1):113–120. 5262992 10.1073/pnas.64.1.113 PMC286134

[20] Smith RE, Horwitz BA. Brown fat and thermogenesis. Physiol Rev. 1969;49(2):330–425. 4888392 10.1152/physrev.1969.49.2.330

[21] Yao L, Heuser-Baker J, Herlea-Pana O, Deficiency in adipocyte chemokine receptor CXCR4 exacerbates obesity and compromises thermoregulatory responses of brown adipose tissue in a mouse model of diet-induced obesity. FASEB J. 2014;28(10): 4534–4550. 25016030 10.1096/fj.14-249797 PMC4202106

[22] Tseng YH, Kriauciunas KM, Kokkotou E, Differential roles of insulin receptor substrates in brown adipocyte differentiation. Mol Cell Biol. 2004;24(5):1918–1929. 14966273 10.1128/MCB.24.5.1918-1929.2004 PMC350563

[23] Bortell N, Najera JA, Sanchez-Alavez M, In vivo effects of methamphetamine on brown fat reactive oxygen species and mitochondria. Temperature. 2015;2(4):453. 10.1080/23328940.2015.1091874 PMC4729381 26835504

[24] Cline MS, Smoot M, Cerami E, Integration of biological networks and gene expression data using Cytoscape. Nat Protoc. 2007;2(10):2366–2382. 17947979 10.1038/nprot.2007.324 PMC3685583

[25] Shannon P, Markiel A, Ozier O, Cytoscape: a software environment for integrated models of biomolecular interaction networks. Genome Res. 2003;13(11):2498–2504. 14597658 10.1101/gr.1239303 PMC403769

[26] Franz M, Rodriguez H, Lopes C, GeneMANIA update 2018. Nucleic Acids Res. 2018;46(W1):W60–W64. 29912392 10.1093/nar/gky311 PMC6030815

[27] Montojo J, Zuberi K, Rodriguez H, GeneMANIA: fast gene network construction and function prediction for Cytoscape. F1000Res. 2014;3:153. 25254104 10.12688/f1000research.4572.1 PMC4168749

[28] Montojo J, Zuberi K, Rodriguez H, GeneMANIA Cytoscape plugin: fast gene function predictions on the desktop. Bioinformatics. 2010;26(22):2927–2928. 20926419 10.1093/bioinformatics/btq562 PMC2971582

[29] Zuberi K, Franz M, Rodriguez H, GeneMANIA prediction server 2013 update. Nucleic Acids Res. 2013;41:W115–W122. 23794635 10.1093/nar/gkt533 PMC3692113

[30] Basova LV, Kesby JP, Kaul M, Systems biology analysis of the antagonizing effects of HIV-1 Tat expression in the brain over transcriptional changes caused by methamphetamine sensitization. Viruses. 2020;12(4):426. 10.3390/v12040426 PMC7232389 32283831

[31] Gersten M, Alirezaei M, Marcondes MC, An integrated systems analysis implicates EGR1 downregulation in simian immunodeficiency virus encephalitis-induced neural dysfunction. J Neurosci. 2009;29(40):12467–12476. 19812322 10.1523/JNEUROSCI.3180-09.2009 PMC2802851

[32] Chouchani ET, Kazak L, Spiegelman BM. Mitochondrial reactive oxygen species and adipose tissue thermogenesis: bridging physiology and mechanisms. J Biol Chem. 2017;292(41): 16810–16816. 28842500 10.1074/jbc.R117.789628 PMC5641863

[33] Cannon B, Nedergaard J. Brown adipose tissue: function and physiological significance. Physiol Rev. 2004;84(1):277–359. 14715917 10.1152/physrev.00015.2003

[34] Cannon B, Nedergaard J. Studies of thermogenesis and mitochondrial function in adipose tissues. Methods Mol Biol. 2008; 456:109–121. 18516556 10.1007/978-1-59745-245-8_8

[35] Barja G Mitochondrial oxygen consumption and reactive oxygen species production are independently modulated: implications for aging studies. Rejuvenation Res. 2007;10(2):215–224. 17523876 10.1089/rej.2006.0516

[36] Garcia MDC, Pazos P, Lima L, Regulation of energy expenditure and brown/beige thermogenic activity by interleukins: new roles for old actors. Int J Mol Sci. 2018;19(9):2569. 10.3390/ijms19092569 PMC6164446 30158466

[37] Van Dyken SJ, Locksley RM. Interleukin-4- and interleukin-13-mediated alternatively activated macrophages: roles in homeostasis and disease. Annu Rev Immunol. 2013;31:317–343. 23298208 10.1146/annurev-immunol-032712-095906 PMC3606684

[38] Jacobsson A, Muhleisen M, Cannon B, The uncoupling protein thermogenin during acclimation: indications for pretranslational control. Am J Physiol. 1994;267(4):R999–R1007. 7943441 10.1152/ajpregu.1994.267.4.R999

[39] Shore AM, Karamitri A, Kemp P, Cold-induced changes in gene expression in brown adipose tissue, white adipose tissue and liver. PLoS One. 2013;8(7):e68933. 23894377 10.1371/journal.pone.0068933 PMC3718809

[40] Brown EL, Hazen BC, Eury E, Estrogen-related receptors mediate the adaptive response of brown adipose tissue to adrenergic stimulation. iScience. 2018;2:221–237. 29888756 10.1016/j.isci.2018.03.005 PMC5993202

[41] Gifford A, Towse TF, Walker RC, Characterizing active and inactive brown adipose tissue in adult humans using PET-CT and MR imaging. Am J Physiol Endocrinol Metab. 2016;311(1): E95–E104. 27166284 10.1152/ajpendo.00482.2015 PMC4967150

[42] Contreras C, Nogueiras R, Dieguez C, Traveling from the hypothalamus to the adipose tissue: the thermogenic pathway. Redox Biol. 2017;12:854–863. 28448947 10.1016/j.redox.2017.04.019 PMC5406580

[43] Rohrer B, Bandyopadhyay M, Beeson C. Reduced metabolic capacity in aged primary retinal pigment epithelium (RPE) is correlated with increased susceptibility to oxidative stress. Adv Exp Med Biol. 2016;854:793–798. 26427491 10.1007/978-3-319-17121-0_106

[44] Yang X, Wang Y, Li Q, The main molecular mechanisms underlying methamphetamine- induced neurotoxicity and implications for pharmacological treatment. Front Mol Neurosci. 2018;11:186. 29915529 10.3389/fnmol.2018.00186 PMC5994595

[45] Park M, Hennig B, Toborek M. Methamphetamine alters occludin expression via NADPH oxidase-induced oxidative insult and intact caveolae. J Cell Mol Med. 2012;16(2):362–375. 21435178 10.1111/j.1582-4934.2011.01320.x PMC3133868

[46] Altomare A, Baron G, Brioschi M, N-acetyl-cysteine regenerates albumin Cys34 by a thiol-disulfide breaking mechanism: an explanation of its extracellular antioxidant activity. Antioxidants. 2020;9(5):367. 10.3390/antiox9050367 PMC7278672 32354002

[47] Ezeriņa D, Takano Y, Hanaoka K, N-acetyl cysteine functions as a fast-acting antioxidant by triggering intracellular H2S and sulfane sulfur production. Cell Chem Biol. 2018;25(4): 447.e4–459.e4. 29429900 10.1016/j.chembiol.2018.01.011 PMC6455997

[48] Mailloux RJ, Adjeitey CN, Xuan JY, Crucial yet divergent roles of mitochondrial redox state in skeletal muscle vs. brown adipose tissue energetics. FASEB J. 2012;26(1):363–375. 21940996 10.1096/fj.11-189639

[49] Vosselman MJ, van der Lans AA, Brans B, Systemic β-adrenergic stimulation of thermogenesis is not accompanied by brown adipose tissue activity in humans. Diabetes. 2012;61(12): 3106–3113. 22872233 10.2337/db12-0288 PMC3501890

[50] Wu J, Cohen P, Spiegelman BM. Adaptive thermogenesis in adipocytes: is beige the new brown? Genes Dev. 2013;27(3): 234–250. 23388824 10.1101/gad.211649.112 PMC3576510

[51] Takekawa M, Saito H. A family of stress-inducible GADD45-like proteins mediate activation of the stress-responsive MTK1/MEKK4 MAPKKK. Cell. 1998;95(4):521–530. 9827804 10.1016/s0092-8674(00)81619-0

[52] Salvador JM, Brown-Clay JD, Fornace AJ Jr. Gadd45 in stress signaling, cell cycle control, and apoptosis. Adv Exp Med Biol. 2013; 793:1–19. 24104470 10.1007/978-1-4614-8289-5_1

[53] Lu G, Xu H, Zhao W, Upregulation of long noncoding RNA Gadd45a is associated with sevoflurane-induced neurotoxicity in rat neural stem cells. Neuroreport. 2018;29(8):605–614. 29521679 10.1097/WNR.0000000000000980

[54] Zhang D, Zhang W, Li D, GADD45A inhibits autophagy by regulating the interaction between BECN1 and PIK3C3. Autophagy. 2015;11(12):2247–2258. 26636486 10.1080/15548627.2015.1112484 PMC4835191

[55] Vairapandi M, Balliet AG, Hoffman B, GADD45b and GADD45g are cdc2/cyclinB1 kinase inhibitors with a role in S and G2/M cell cycle checkpoints induced by genotoxic stress. J Cell Physiol. 2002;192(3):327–338. 12124778 10.1002/jcp.10140

[56] Li X, Marshall PR, Leighton LJ, The DNA repair-associated protein Gadd45gamma regulates the temporal coding of immediate early gene expression within the prelimbic prefrontal cortex and is required for the consolidation of associative fear memory. J Neurosci. 2019;39(6):970–983. 30545945 10.1523/JNEUROSCI.2024-18.2018 PMC6363930

[57] Gantner ML, Hazen BC, Conkright J, GADD45γ regulates the thermogenic capacity of brown adipose tissue. Proc Natl Acad Sci USA. 2014;111(32):11870–11875. 25071184 10.1073/pnas.1406638111 PMC4136592

[58] Gantner ML, Hazen BC, Eury E, Complementary roles of estrogen-related receptors in brown adipocyte thermogenic function. Endocrinology. 2016;157(12):4770–4781. 27763777 10.1210/en.2016-1767 PMC5133354

